# Effects of C and Al Alloying on Constitutive Model Parameters and Hot Deformation Behavior of Medium-Mn Steels

**DOI:** 10.3390/ma17030732

**Published:** 2024-02-03

**Authors:** Guangshun Guo, Mingming Wang, Hongchao Ji, Xiaoyan Zhang, Dongdong Li, Chenyang Wei, Fucheng Zhang

**Affiliations:** 1 College of Mechanical Engineering, North China University of Science and Technology, Tangshan 063210, China; 2 School of Materials Science and Engineering, Zhejiang University, Hangzhou 310030, China; 3 College of Metallurgy and Energy, North China University of Science and Technology, Tangshan 063210, China; 4 State Key Laboratory of Metastable Materials Science and Technology, Yanshan University, Qinhuangdao 066004, China

**Keywords:** medium-Mn steel, hot deformation behavior, constitutive model, processing map

## Abstract

Single-pass isothermal hot compression tests on four medium-Mn steels with different C and Al contents were conducted using a Gleeble-3500 thermal simulation machine at varying deformation temperatures (900–1150 °C) and strain rates (0.01–5 s^−1^). Based on friction correction theory, the friction of the test stress–strain data was corrected. On this basis, the Arrhenius constitutive model of experimental steels considering Al content and strain compensation and hot processing maps of different experimental steels at a strain of 0.9 were established. Moreover, the effects of C and Al contents on constitutive model parameters and hot processing performance were analyzed. The results revealed that the increase in C content changed the trend of the thermal deformation activation energy *Q* with the true strain. The *Q* value of 2C7Mn3Al increased by about 50 KJ/mol compared with 7Mn3Al at a true strain greater than 0.4. In contrast, increasing the Al content from 0 to 1.14 wt.% decreased the activation energy of thermal deformation in the true strain range of 0.4–0.9. Continuing to increase to 3.30 wt.% increased the Q of 7Mn3Al over 7Mn by about 65 KJ/mol over the full strain range. In comparison, 7Mn1Al exhibited the best hot processing performance under the deformation temperature of 975–1125 °C and strain rate of 0.2–5 s^−1^. This is due to the addition of C element reduces the δ-ferrite volume fraction, which leads to the precipitation of κ-carbides and causes the formation of microcracks; an increase in Al content from 0 to 1.14 wt.% reduces the austenite stability and improves the hot workability, but a continued increase in the content up to 3.30 wt.% results in the emergence of δ-ferrite in the microstructure, which slows down the austenite DRX and not conducive to the hot processing performance.

## 1. Introduction

Environmental protection, weight reduction, and improved safety stand as the primary requirements within the automotive industry. One of the means to achieve lightweight automobiles is the utilization of advanced high-strength steels. As an important component in third-generation advanced high-strength steels, medium-Mn steel has attracted considerable attention owing to its multiphase, multi-scale, and metastable characteristics [1,2]. The ultra-fine dual-phase microstructure of medium-Mn steel, obtained through intercritical annealing treatment, is accompanied by a large amount of retained austenite [3,4,5,6]. Transformation-induced plasticity (TRIP) is a major strengthening and toughening mechanism for medium-Mn steels. During plastic deformation, austenite grains possessing appropriate stability transform into martensite, thereby delaying crack nucleation and micropore aggregation. Consequently, medium-Mn steels exhibit higher mechanical properties [7]. The microstructure heredity during the preparation of medium-Mn steel affects its final microstructure and mechanical properties. Therefore, modifying the initial microstructure before annealing by optimizing the hot deformation process parameters is a crucial method for controlling the mechanical properties of medium-Mn steels. However, current research mainly focuses on the relationship between microstructure mechanical properties, the austenite reverse phase transformation mechanism, and the stability of austenite medium-Mn steels [5,8,9,10]. Studies on flow stress, dynamic recovery (DRV), and dynamic recrystallization during hot deformation and the effects of alloying elements on constitutive model parameters and hot-forming properties of steels remain to be explored further.

Upon introducing the Zener–Hollomon parameter, Wan et al. [11] established the dynamic recrystallization (DRX) flow stress model for Fe-Mn-C-Al steel and investigated the optimal hot working parameters to achieve a uniform fine microstructure. The occurrence of dynamic recrystallization behavior in the steel became challenging with an increase in the Z parameters. Li et al. [12] studied the DRX characteristics of Fe-8Mn-6Al-0.2C steel and analyzed its microstructure evolution qualitatively. Their findings indicated that elevated temperatures and reduced strain rates can effectively promote the growth of austenite and α-ferrite, respectively. Liu et al. [13] analyzed the deformation microstructure of Fe-Mn-C-Al dual-phase steel during the DRX process and further verified its microstructure characteristics by combining it with the hot processing map. Based on the DRX behavior and microstructure evolution of medium-Mn steel, Sun et al. [14] established a strain-compensated constitutive model and the grain size evolution model. Their findings suggested that the flow stress–strain curve showed evident positive deformation temperature and negative strain rate sensitivity. The recrystallized grain size gradually increased with the deformation temperature and strain rate, and the size distribution range was expanded considerably. Li et al. [15] delineated how δ-ferrite and austenite affected the flow stress characteristics of Fe-Mn-C-Al lightweight steel during the early and late stages of compression–deformation, respectively. Li et al. established the austenite DRX kinetic model without considering DRV and δ-ferrite DRX. They also highlighted that the growth of austenite grains at low strain rates promoted the transformation of δ-ferrite from a banded structure to an island structure. In addition, various scholars have explored the effects of alloying elements on the hot workability of medium-Mn steels. The increase in Ti content reduces the deformation activation energy, decreases the original austenite grain size, accelerates the occurrence of the DRX process, and promotes the formation of fine lath martensite microstructure [16]. Krbat’a et al. [17] investigated the high-temperature plastic behavior of low manganese Si-containing medium-Mn steel. They analyzed the effects of deformation temperature on peak stress via flow curve and discussed the phase transformation of the steel from the perspective of DRV and DRX.

The aforementioned research mainly examined the effects of hot deformation process parameters and alloy elements on the flow stress and microstructure, but the application of medium-Mn steels from sheet to automobile parts involves a complex hot forming process. Therefore, it is necessary to study the effects of C and Al content on the constitutive model parameters, hot processing map, and microstructure after deformation of medium-Mn steels and obtain the optimal forming process interval and variation rule of medium-Mn steels with different element content, so as to provide theoretical guidance for the numerical simulation and practical application of medium-Mn steels as auto parts. In this study, the high-temperature plastic deformation behavior of medium-Mn steels was studied by single-pass hot compression tests. The flow stress, under varying deformation conditions, was friction-corrected, and a constitutive model of the steels considering the content of Al was established. The effects of C and Al contents on the constitutive model parameters and hot deformation behavior of medium-Mn steel were studied. The relationship between element content and microstructure was analyzed by selecting deformed samples with different hot working conditions in combination with hot processing maps.

## 2. Materials and Methods

**Figure 1:**
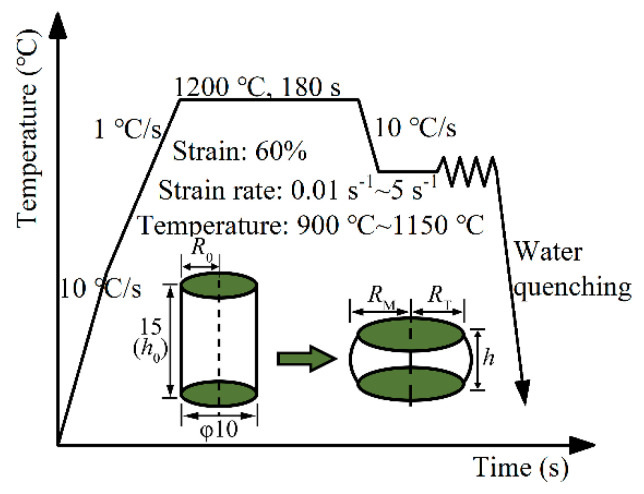
Schematic diagram of the isothermal hot compression test process.

Steels were smelted in a vacuum furnace and cast into Φ200 mm ingots. These ingots were heated to 1200 °C for 2 h and subsequently hot forged into 50 mm × 50 mm billets. Square-shaped specimens, with 15 mm × 15 mm × 15 mm dimensions, were processed from the forging billet. After the samples were ground, their chemical composition was tested using SparkCCD 7000 optical emission spectrometer (OES, NCS Testing Technology Co., Ltd., Beijing, China). Table 1 displays the chemical composition of four kinds of 7Mn steels with varying C and Al contents. Cylindrical samples, with dimensions Φ10 mm × 15 mm, were processed longitudinally from the forging billet using wire-cut electrical discharge machining (Suzhou New Spark Machine Tool Co., Ltd., Suzhou, China), and the circumference and end face of specimens were polished to ensure that the surface roughness was less than 0.4 μm. Isothermal hot compression tests were conducted on a Gleeble-3500 thermal simulator at six temperatures (900, 950, 1000, 1050, 1100, and 1150 °C) and four strain rates (0.01, 0.1, 1, and 5 s^−1^). Samples were heated to 600 °C at a rate of 10 °C/s, then heated to 1200 °C at a rate of 1 °C/s and held for 180 s to homogenize the microstructure. Subsequently, they were cooled to the deformation temperature at a rate of 10 °C/s and held for 30 s to eliminate the temperature gradient before compression tests to ensure homogenization of the entire sample temperature. Samples were compressed according to the predetermined strain of 60%. Immediate water quenching was performed after compression to retain the high-temperature deformed microstructure. The hot deformation process is shown in Figure 1. Deformed samples were cut through the center parallel to the compression axis for microstructure observations. After the center sections were ground, polished, and etched in a 4% nitric acid alcohol solution (volume fraction) for 10 s, the microstructures were observed using a Scios scanning electron microscope (SEM, Thermo Fisher Scientific (CHINA) Co., Ltd., Shanghai, China) at 20 kV and 0.4 nA.

## 3. Results

### 3.1. Thermodynamic Calculation

**Figure 2:**
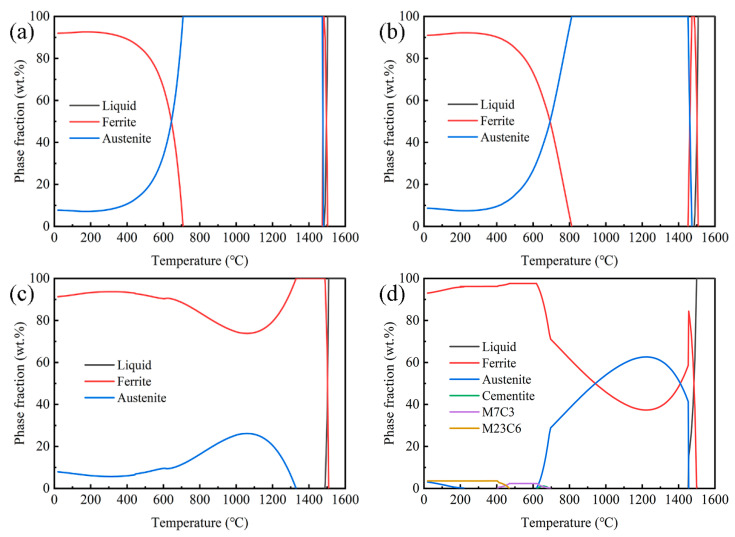
Equilibrium phase diagrams of steels: (**a**) 7Mn, (**b**) 7Mn1Al, (**c**) 7Mn3Al, and (**d**) 2C7Mn3Al.

Based on the chemical composition of experimental steels in Table 1, the volume fraction of each phase in the equilibrium microstructure at various temperatures was calculated using the General Steel module of JMatPro 7.0 software. The start and end temperatures are 20 °C and 1600 °C, respectively, and the step is 10 °C. Figure 2 shows the results of the calculations. A comparison between 7Mn and 7Mn1Al reveals that with an increase in Al content from 0 to 1.14 wt.%, the phase diagram shapes remain similar, but the transformation temperatures alter. The complete austenitizing temperatures (A_3_) rise from 708 °C to 812 °C, and the temperatures for the same mass fraction of the two phases increase from 645 °C to 694 °C. This change reduces the high-temperature processing range for the steels. At room temperature, the microstructure of both steels comprises ferrite and austenite, displaying minimal differences in phase content, as depicted in Figure 2a,b. Upon increasing the Al content to 3.3 wt.%, notable changes occur in the phase diagram compared with the previous compositions. Above 1400 °C, the liquid phase transforms into δ-ferrite, but an immediate transformation into austenite does not occur. At 1329 °C, some δ-ferrite transitions into austenite, reaching its maximum content around 1060 °C. Subsequently, austenite starts transforming into α-ferrite, as indicated in Figure 2c. Notably, δ-ferrite persists, owing to the stabilizing effect of the Al element through solid solution strengthening, enabling its retention at room temperature [18]. Comparing 7Mn3Al and 2C7Mn3Al, it becomes evident that with an increase in C content to 0.2 wt.%, the liquid phase initially transforms into δ-ferrite at high temperatures. At 1455 °C, the δ-ferrite content peaks at 84.4 wt.%, then transitions into austenite. At approximately 1220 °C, the austenite content reaches a maximum of 62.6 wt.%. Carbides begin precipitating around 690 °C, as depicted in Figure 2d.

An increase in Al content reduces the austenite content at the selected deformation temperature range, whereas an increase in C content elevates the austenite content within the same temperature range. This aligns with the established understanding that Al and C elements tend to stabilize ferrite and austenite, respectively. Specifically, in the temperature range of 900 °C to 1150 °C, the microstructure of 7Mn and 7Mn1Al consists solely of single-phase austenite. Conversely, 7Mn3Al and 2C7Mn3Al exhibit a dual-phase microstructure of austenite and ferrite within this range, albeit with differing phase proportions. The former primarily comprises ferrite, whereas the latter is predominantly composed of austenite.

### 3.2. Hot Deformation Flow Stress Curve and Friction-Correction

During the hot compression process, the sample undergoes friction from the equipment’s indenter, causing metal accumulation along the flow direction. This phenomenon, known as “bulging”, leads to a certain deviation between the experimental and actual values, as depicted in Figure 1. To enhance accuracy, the high-temperature flow stress is adjusted or corrected to refine test data.

In general, the following relationship between the stress before and after friction correction is expressed as follows [19]:

(1) $$\frac{\sigma_{0}}{\sigma }=\frac{8bR}{H}\left\{{\left[\frac{1}{12}+{\left(\frac{H}{Rb}\right)}^{2}\right]}^{\frac{3}{2}}-{\left(\frac{H}{Rb}\right)}^{3}-\frac{me^{-\frac{b}{2}}}{24\sqrt{3}\left(e^{-\frac{b}{2}}-1\right)}\right\}$$

where *σ*_0_ is the original flow stress data (MPa); *σ* is the flow stress after friction-correction (MPa); *b* is the drum belly parameter; *R* and *H* are the instantaneous radius and height in the hot compression process (mm), respectively; *m* is the friction factor; and e is the natural constant.

*b*, *R*, *H*, and *m* can be calculated by Equations (2)–(5), respectively:

(2) $$b=4\times \frac{R_{M}-R_{T}}{R_{f}}\times \frac{h}{h_{0}-h}$$

(3) $$R=R_{0}e^{\frac{\varepsilon }{2}}$$

(4) $$H=h_{0}e^{-\varepsilon }$$

(5) $$m=\frac{\left(R_{f}/H\right)b}{\left(4/\sqrt{3}\right)-\left(2b/3\sqrt{3}\right)}$$

where *R*_M_ is the radius of the bulging area after compression of the sample (mm); *R*_T_ is the radius of the end face of the sample after compression (mm); *R*_f_ is the average radius of the sample after compression (mm); *h* is the height of the sample after compression (mm); *h*_0_ is the original height of the sample (mm); *R*_0_ is the original radius of the sample (mm); and *ε* is the strain of the sample.

(6) $$R_{T}=\sqrt{3\times \frac{h_{0}}{h}\times R_{0}^{2}-2R_{M}^{2}}$$

(7) $$R_{f}=R_{0}\sqrt{\frac{h_{0}}{h}}$$

**Figure 3:**
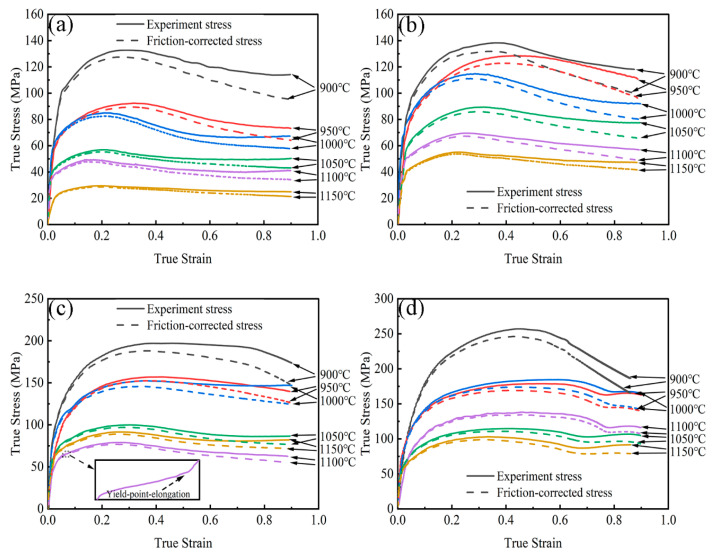
True stress–true strain curves of 7Mn3Al at various deformation parameters: (**a**) 0.01 s^−1^, (**b**) 0.1 s^−1^, (**c**) 1 s^−1^, and (**d**) 5 s^−1^.

The true stress-true strain curves of 7Mn3Al before and after correction at varying temperatures are shown in Figure 3. Post-friction-correction, the curves exhibit general consistency with the pre-correction curves at lower strain levels. However, as the strain increases, the disparity between the two curves at the same deformation temperature progressively grows. This occurrence arises from the initial stages of hot compression–deformation, where the contact area between the sample’s end face and the indenter is minimal, resulting in minimal frictional influence on metal flow. Consequently, the two curves remain largely similar. However, with increasing strain, the end face area enlarges, intensifying friction between the surfaces, thereby augmenting the metal’s flow resistance. Ultimately, this contributes to a more pronounced difference between the pre and post-correction curves.

At the onset of deformation, dislocation multiplication and dislocation glide predominantly govern the process, leading to pronounced work hardening in the initial stages. Consequently, the flow stress experiences rapid escalation with increasing strain, reaching a peak swiftly. Subsequent to this initial deformation-strengthening phase driven by work hardening, the nucleation and growth of dynamically recrystallized grains play a crucial role. This phase allows for the dissipation of deformation energy and the absorption of defects, such as dislocations and sub-grain boundaries formed during the deformation process. As a result, a softening effect emerges, diminishing the rate of work hardening and leading to a gradual decline in flow stress [20,21]. Eventually, a dynamic equilibrium is attained when the mechanisms of work hardening and dynamic softening reach a balance. At this stage, the flow stress tends to stabilize [22].

The flow stress curve is significantly influenced by deformation temperature and strain rate. Elevated temperature increases the thermal vibration amplitude of metal atoms, thereby reducing dislocation motion resistance. Consequently, an increased number of slip systems become activated, promoting grain boundary migration and enhancing DRX. Simultaneously, higher deformation temperatures augment the driving force for vacancy atom diffusion, dislocation climb, and cross slip, favoring the occurrence of softening mechanisms. In contrast, higher strain rates result in shorter deformation, grain boundary migration, and dislocation reconstruction times. This shorter duration delays the onset of dynamic recrystallization, leading to abbreviated dynamic recrystallization times and predominantly manifesting DRV, resulting in a lesser softening effect [23]. Conversely, lower strain rates provide more time for DRX and DRV during deformation, intensifying the material’s softening effect. Consequently, the material exhibits a lower flow stress value under slower strain rate conditions. Hence, flow stress curves demonstrate two distinct types: the DRX type curve, featuring pronounced softening effects at lower strain rates, and the DRV type curve, displaying less obvious softening effects at higher strain rates.

DRX flow stress curves can be categorized into three types: single-peak, multi-transient steady-state, and multi-peak [24]. As depicted in Figure 3d, at a lower temperature of 900 °C and a higher strain rate of 5 s^−1^, the curve exhibits a single peak characteristic, with the flow stress declining after reaching its maximum value. This characteristic curve pattern is commonly observed in metals with high stacking fault energy [25]. Conversely, the occurrence of stress fluctuation with multiple peaks, known as multi-peak behavior, can be observed at lower strain rates, such as at a temperature of 900 °C and a strain rate of 0.01 s^−1^, illustrated in Figure 3a. This behavior is attributable to the presence of multiple independent DRX cycles [24].

A yield-point elongation is observable on the flow stress curve at the initial stage of deformation under the deformation conditions of 1100 °C and a strain rate of 1 s^−1^, as highlighted in the enlarged section of Figure 3c. This phenomenon arises from the uneven distribution of ferrite and austenite during deformation [26]. Due to the differing yield strengths of the two phases, deformation initiates in the softer ferrite phase. As deformation progresses, a multitude of dislocations proliferates and becomes entangled within the ferrite, resulting in work hardening. Consequently, the strain is subsequently transferred to the surrounding austenite, leading to the observation of two yield points—an occurrence known as yield-point elongation [26,27].

### 3.3. Establishment and Analysis of Constitutive Model

Metal materials exhibit complex rheological properties due to the influence of deformation temperature, strain rate, and strain during hot deformation. To describe the relationship between different factors, the constitutive relationship of steel was established using the Zener–Hollomon parameter [28]. The corresponding equations are as follows:

(8) $$\dot{\varepsilon }=Af(\sigma )\exp\left(-\frac{Q}{RT}\right)$$

(9) $$Z=\dot{\varepsilon }\exp\left(\frac{Q}{RT}\right)$$

where $\dot{\varepsilon }$ is the strain rate (s^−1^); *A* is the material constant; *f*(*σ*) is the function of stress; *Q* is the activation energy (J/mol); *R* is the molar gas constant (8.314 J·mol^−1^·K^−1^); and *T* is the thermodynamic temperature (K). The Zener–Hollomon parameter is generally considered a function of flow stress and is divided into three equations according to different stress levels [29]:

(10) $$f(\sigma )=\left\{\begin{array}{ll} \sigma^{n_{1}} & \alpha \sigma <0.8\;(\mathrm{power}\;\mathrm{function})\\ \exp(\beta \sigma ) & \alpha \sigma >1.2\;(\mathrm{exponential}\;\mathrm{function})\\ {\left[\sinh(\alpha \sigma )\right]}^{n} & \mathrm{for}\;\mathrm{all}\;\sigma \;(\mathrm{hyperbolic}\;\mathrm{sine}\;\mathrm{function}) \end{array}\right.$$

where *n*_1_, *n*, *α*, and *β* are material constants, *α* = *β*/*n*_1_.

The hyperbolic sine function that describes the entire stress level is used as the constitutive model to describe the variation in the flow stress with temperature and strain rate during the hot deformation of steels [30]:

(11) $$Z=\dot{\varepsilon }\exp\left(\frac{Q}{RT}\right)=A{\left[\sinh(\alpha \sigma )\right]}^{n}$$

Substituting Equation (10) into Equation (8), the logarithm on both sides is taken, and the partial derivative is calculated to obtain:

(12) $$n_{1}=\frac{\partial \ln\dot{\varepsilon }}{\partial \ln\sigma }$$

(13) $$\beta =\frac{\partial \ln\dot{\varepsilon }}{\partial \sigma }$$

When the temperature *T* or $\dot{\varepsilon }$ are constant, the logarithm of two sides of Equation (11) and the partial derivative can be obtained:

(14) $$n={\left[\frac{\partial \ln\dot{\varepsilon }}{\partial \ln\left[\sinh(\alpha \sigma )\right]}\right]}_{T}$$

(15) $$\frac{Q}{nR}={\left[\frac{\partial \ln\left[\sinh(\alpha \sigma )\right]}{\partial (1/T)}\right]}_{\dot{\varepsilon }}$$

Four parameters (*n*_1_, *β*, *n*, and *Q/nR*) of the peak stress constitutive model can be obtained by linear regression of ln$\dot{\varepsilon }$ − ln*σ*, ln$\dot{\varepsilon }$ − *σ*, ln$\dot{\varepsilon }$ − ln[sinh(*ασ*)], and ln[sinh(*ασ*)] − 1/*T* under different hot working conditions to obtain the average slope value, and then *α* can be calculated by the equation of *α* = *β*/*n*_1_.

**Figure 4:**
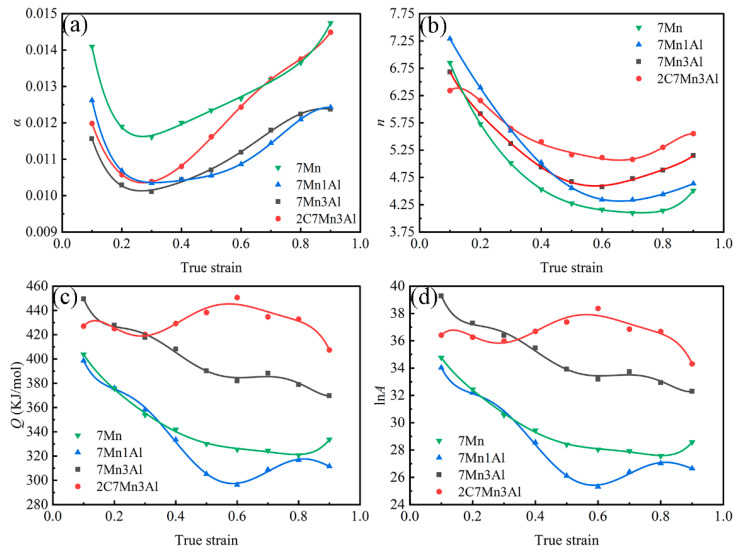
Relationship between (**a**) *α*, (**b**) *n*, (**c**) *Q*, (**d**) ln*A* and true strain of steels.

The parameters *α*, *n*, and *Q*/*nR* are introduced into Equation (11) under constant deformation temperature *T* and strain rate $\dot{\varepsilon }$ to determine *A*. Upon leveraging the properties of the hyperbolic sine function and the correlation between peak stress and deformation temperature, the Arrhenius peak stress constitutive model for the steel can be established through Equations (8) and (10). However, this model solely accounts for the impact of deformation temperature and strain rate on deformation resistance. Studies have revealed that under low-temperature conditions, deformation resistance is significantly influenced by strain [14,31]. Consequently, to accurately predict flow stress across the entire deformation range, *α*, *n*, *Q*, and *A* are fitted as polynomial functions of strain using a strain-compensation method. Figure 4 illustrates the relationship between the four parameters of the constitutive model and the true strain *ε*.

The stress level parameter *α* varies with material and deformation conditions [32]. Figure 4a shows that the stress level parameters of the four steels decrease to the lowest point and then increase gradually with an increase in strain. Comparing 7Mn, 7Mn1Al, and 7Mn3Al reveals that the addition of the Al element reduces the stress levels under the same strain in the absence of the C element. However, 7Mn1Al and 7Mn3Al exhibit alternating changes. Specifically, 7Mn1Al steel exhibits higher stress levels when the strain is less than 0.4, while 7Mn3Al demonstrates higher stress levels when the strain exceeds 0.4. Further comparison between 7Mn3Al and 2C7Mn3Al demonstrates that under the same Al content, the stress level parameters increase with a rise in C content under equivalent strains. This difference becomes progressively evident, particularly when the strain exceeds 0.3.

The stress index, represented by *n*, initially decreases and then increases with an increase in strain. Additionally, the stress index exhibits an upward trend with an increase in Al content. Specifically, the stress index of 7Mn1Al steel surpasses that of 7Mn3Al when the strain is below 0.4 but falls below that of 7Mn3Al when the strain exceeds 0.4. Furthermore, as the C content increases, the stress index of 2C7Mn3Al demonstrates higher values compared with the three other steels when the strain exceeds 0.3.

The thermal deformation activation energy *Q* characterizes the energy barrier that dislocations must surpass during thermal deformation, signifying the processing performance of the alloy. Figure 4c illustrates that, except for 2C7Mn3Al, the hot deformation activation energy of other steels decreases with an increase in deformation level. Specifically, for 7Mn1Al, the activation energy decreases initially, then increases, and subsequently decreases again, peaking at 0.6 strain. Comparing the thermal deformation activation energy *Q* between 7Mn and 7Mn3Al, as the Al content increases from 0 to 3 wt.%, the thermal activation energy of the steel increases under the same strain. Similarly, comparing the thermal deformation activation energy *Q* between 7Mn3Al and 2C7Mn3Al, the alteration in activation energy demonstrates a similar trend when the strain is below 0.3. However, as strain increases, the former exhibits a declining trend, while the latter initially rises and subsequently declines. Based on this analysis, an increase in Al and C content can enhance the thermal deformation activation energy under specific strain conditions.

**Figure 5:**
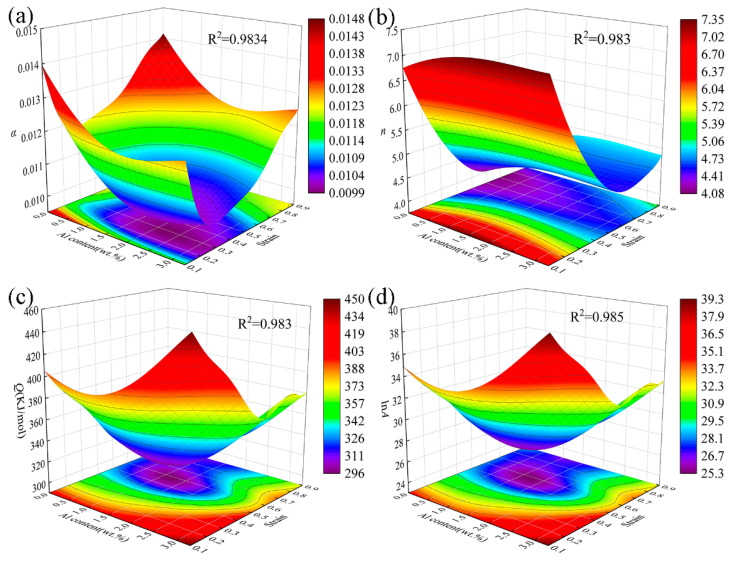
Related parameter fitting diagram: (**a**) *α*, (**b**) *n*, (**c**) *Q*, and (**d**) ln*A*.

The material’s constitutive model parameters, considering the Al content and strain-compensation, are derived by taking a matrix of the material model parameters across various aluminum contents and multiple strain conditions, followed by nonlinear surface fitting. The fitting outcomes for different parameters are depicted in Figure 5.

The connection between the constitutive model parameters and strain can be articulated through a polynomial function encompassing the aluminum content and strain, as demonstrated in Equation (16). Table 2 provides the coefficients of the fitting polynomials for various parameters.

(16) $$G=k_{0}+a_{1}w+a_{2}w^{2}+a_{3}w^{3}+a_{4}w^{4}+b_{1}\varepsilon +b_{2}\varepsilon^{2}+b_{3}\varepsilon^{3}+b_{4}\varepsilon^{4}+b_{5}\varepsilon^{5}+b_{6}\varepsilon^{6}+b_{7}\varepsilon^{7}$$

where *G* represents different material parameters (*α*, *n*, *Q*, and ln*A*); *k*_0_, *a*_1_…*a*_4_, and *b*_1_…*b*_7_ represent polynomial coefficients; *w* represents the content of Al element (wt.%); *ε* is strain.

Upon analyzing the trends in material parameters, it is possible to determine the flow stress under specific strain conditions. The Arrhenius constitutive model for medium-Mn steels with varying aluminum content under different strain conditions can be formulated as follows:

(17) $$\sigma =\frac{1}{\alpha }\ln\left\{{\left(\frac{Z}{A}\right)}^{\frac{1}{n}}+{\left[{\left(\frac{Z}{A}\right)}^{\frac{2}{n}}+1\right]}^{\frac{1}{2}}\right\}$$

**Figure 6:**
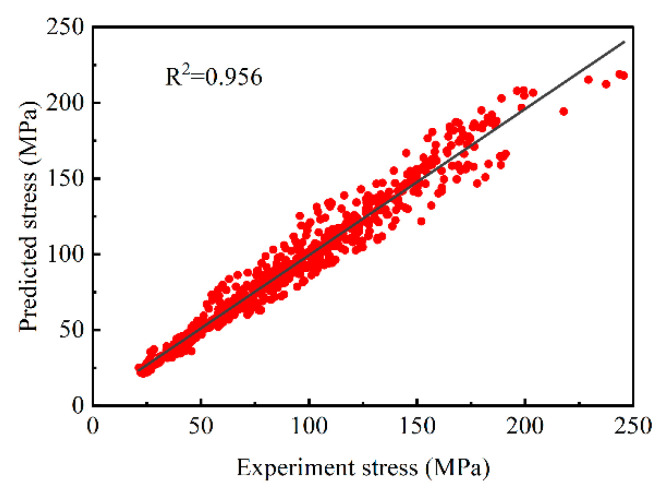
Comparison of predicted values and experimental values under different conditions.

To validate the accuracy of the Arrhenius constitutive model for medium-Mn steels with different Al contents, the predicted outcomes under different Al contents and deformation conditions were compared with experimental results. Figure 6 displays the fitting results of both sets under different conditions. To further assess the model’s precision, the correlation coefficient *R* and the average absolute relative error AARE (%) are utilized to characterize the model’s performance.

(18) $$R=\frac{\sum_{i=1}^{N}\left(\sigma_{Ei}-{\bar{\sigma }}_{E})(\sigma_{Pi}-{\bar{\sigma }}_{Pi}\right)}{\sqrt{\sum_{i=1}^{N}{\left(\sigma_{Ei}-{\bar{\sigma }}_{E}\right)}^{2}\sum_{i=1}^{N}{\left(\sigma_{Pi}-{\bar{\sigma }}_{Pi}\right)}^{2}}}$$

(19) $$AARE=\frac{1}{N}\sum_{i=1}^{N}\left|\frac{\sigma_{Ei}-\sigma_{Pi}}{\sigma_{Ei}}\right|$$

where *N* is the total number of data; $\sigma_{Ei}$ and ${\bar{\sigma }}_{E}$ represent the test value and its average value (MPa), respectively; and $\sigma_{Pi}$ and ${\bar{\sigma }}_{Pi}$ represent the predicted value and its average value (MPa), respectively.

Figure 6 depicts that the predicted value and the experimental value are basically near the straight line, indicating that the stress values of the two are basically the same under different conditions. Calculation shows that *R* = 0.956, AARE = 9.781%, less than 10%.

### 3.4. Establishment and Analysis of Hot Processing Map of Steels

Hot processing maps and instability maps play remarkable roles in investigating constitutive behavior and optimizing process parameters during material thermal deformation processes. Utilizing the DMM, the deformation process is perceived as a nonlinear energy dissipation system. This dissipation primarily comprises plastic deformation energy dissipation (G) and microstructure evolution energy dissipation (*J*) [30]. Consequently, the total energy (*P*) of the external input system can be mathematically expressed as follows:

(20) $$P=G+J=\int_{0}^{\sigma }\dot{\varepsilon }d\sigma +\int_{0}^{\dot{\varepsilon }}\sigma d\dot{\varepsilon }$$

The two energy ratios can be expressed by the strain rate sensitivity index *m*:

(21) $$m={\left(\frac{\partial J}{\partial G}\right)}_{\varepsilon ,T}=\frac{\partial P}{\partial G}\frac{\partial J}{\partial P}=\frac{\sigma d\dot{\varepsilon }}{\dot{\varepsilon }d\sigma }={\left[\frac{\partial \ln\sigma }{\partial \ln\dot{\varepsilon }}\right]}_{\varepsilon ,T}$$

When the energy dissipation process conforms to an ideal linear dissipation pattern, the value of *J* reaches its maximum, and *m* is 1. The power dissipation efficiency *η* is commonly employed to illustrate the correlation between microstructure evolution and energy dissipation, as follows:

(22) $$\eta =\frac{J}{J_{\max}}=\frac{2m}{m+1}$$

The power dissipation efficiency is dependent on the deformation temperature and strain rate. Across a specified strain level, the variation in power dissipation efficiency concerning temperature (*T*) and strain rate constitutes the power dissipation diagram. Regions where the *η* value exceeds 0.3 signify stable microstructure alterations, indicating good plasticity. However, the instability zone may also exhibit high *η* values; hence, relying solely on the power dissipation coefficient *η* for analyzing hot working performance might not be adequate. To assess the material’s behavior comprehensively, the plastic instability criterion to construct a hot processing map for further analysis must be integrated.

The Prasad instability criterion, founded on the DMM and Ziegler’s plastic flow theory, serves to evaluate rheological instability in hot compression scenarios. The specific form of this criterion is expressed as follows:

(23) $$\xi_{p}\left(\dot{\varepsilon },T\right)=\frac{\partial \ln(\frac{m}{m+1})}{\partial \ln\dot{\varepsilon }}+m<0$$

where *ξ*_p_ is a function of strain rate and deformation temperature, the negative value indicates that the material has flow instability and is not suitable for processing within the corresponding process parameters.

**Figure 7:**
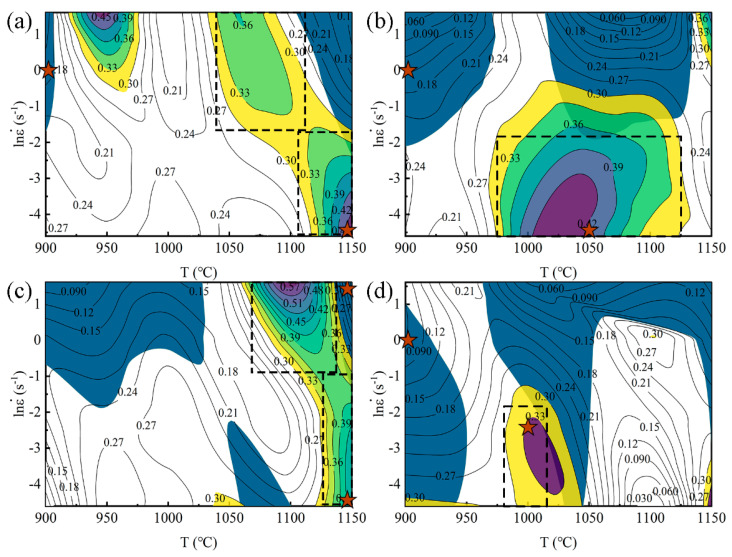
Hot processing map of the steels at 0.9 strain: (**a**) 7Mn, (**b**) 7Mn1Al, (**c**) 7Mn3Al, and (**d**) 2C7Mn3Al. The color-altered region indicates a high-power dissipation efficiency (*η* value), while the blue area signifies instability. Red stars represent the process conditions of samples for microstructure characterization.

The hot processing map is generated upon overlaying the power dissipation map and the rheological instability map. Figure 7 illustrates the hot processing map for various steels under a strain of 0.9. In this figure, the color-altered region indicates a high-power dissipation efficiency (*η* value), while the blue area signifies instability. A comparison between 7Mn and 7Mn1Al reveals that increasing the Al content from 0 to 1.14 wt.% expands the instability zone, primarily concentrated within the temperature range of 900–950 °C and 1025–1125 °C, under strain rates from 0.1–5 s^−1^. Moreover, the broader high-power dissipation zone transforms from a narrow strip to a block-shaped area. Although the area expands, the peak value decreases from 0.45 to 0.42. Conversely, when the Al content increases from 1.14 wt.% to 3.30 wt.%, the instability zone in the temperature range of 900–950 °C widens to 900–1025 °C. Additionally, the higher power dissipation zone shifts to a narrow strip. However, the peak value of 7Mn steel increases from 0.45 to 0.57. This observation suggests that within these corresponding hot working conditions, DRX occurs readily, reaching maximum levels at the peak value of *η*, leading to material flow softening. Consequently, incorporating an appropriate amount of Al enhances hot workability.

Upon comparing 7Mn3Al and 2C7Mn3Al, the low-temperature instability zone (within the deformation temperature range of 1060 °C to 1140 °C and strain rates of 0.4 s^−1^ to 5 s^−1^) shifts from high to low strain rates. Additionally, the area of the instability zone decreases as the C content escalates from 0.03 wt.% to 0.2 wt.%. In contrast to 7Mn3Al, the region where the power dissipation coefficient of 2C7Mn3Al exceeds 0.3 shrinks, and the peak value decreases from 0.57 to 0.33. This reduction suggests an environment less conducive to dynamic recrystallization. Simultaneously, within the temperature range of 1075–1125 °C and strain rates from 0.01–0.1 s^−1^, although the instability parameter surpasses zero, the power dissipation coefficient remains below 0.15. This result indicates that the potential presence of an adiabatic shear band within the sample’s microstructure formed under these conditions causes localized plastic deformation during material deformation. The localized heat generated might not dissipate quickly enough to the surrounding area, resulting in decreased local flow stress. The formation of the shear band consumes substantial energy, leading to a low power dissipation coefficient, accompanied by remarkable local shear deformation. Typically, this region may exhibit crack formation. Once cracks emerge, they tend to propagate along the direction of the shear band, resulting in transgranular cracking during the deformation process. Consequently, the increase in C content appears to worsen thermal processing performance. This finding aligns with the observed trend of hot deformation activation energy *Q* concerning the C and Al element content under a strain of 0.9.

The optimum hot working range for steels is represented by the black dashed wireframes in Figure 7. For 7Mn, the deformation temperature ranges from 1040 °C to 1110 °C, with a strain rate of 0.2 s^−1^ to 5 s^−1^. Additionally, the deformation temperature ranges from 1105 °C to 1150 °C, with a strain rate of 0.01 s^−1^ to 0.2 s^−1^. As for 7Mn1Al steel, the deformation temperature lies between 975 °C and 1125 °C, with a strain rate of 0.2 s^−1^ to 5 s^−1^. In the case of 7Mn3Al, the deformation temperature ranges from 1060 °C to 1140 °C, with a strain rate of 0.4 s^−1^ to 5 s^−1^, and from 1125 °C to 1150 °C, with a strain rate of 0.01 s^−1^ to 0.4 s^−1^. Finally, the deformation temperature for 2C7Mn3Al is between 980 °C and 1120 °C, and the strain rate varies from 0.01 s^−1^ to 0.2 s^−1^. The comparison shows that the optimum hot working interval area of 7Mn1Al is the largest, and the distribution is continuous. Hence, its hot working performance is the best.

### 3.5. Microstructure Evolution

**Figure 8:**
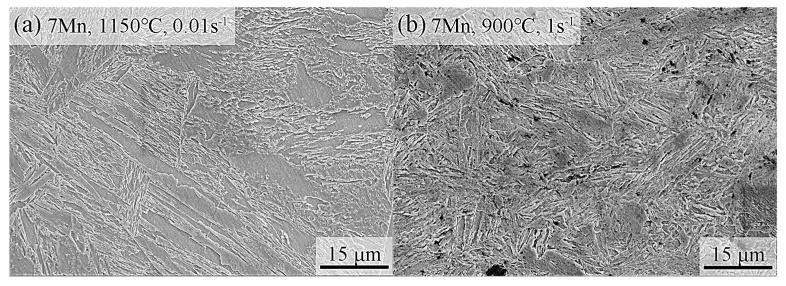

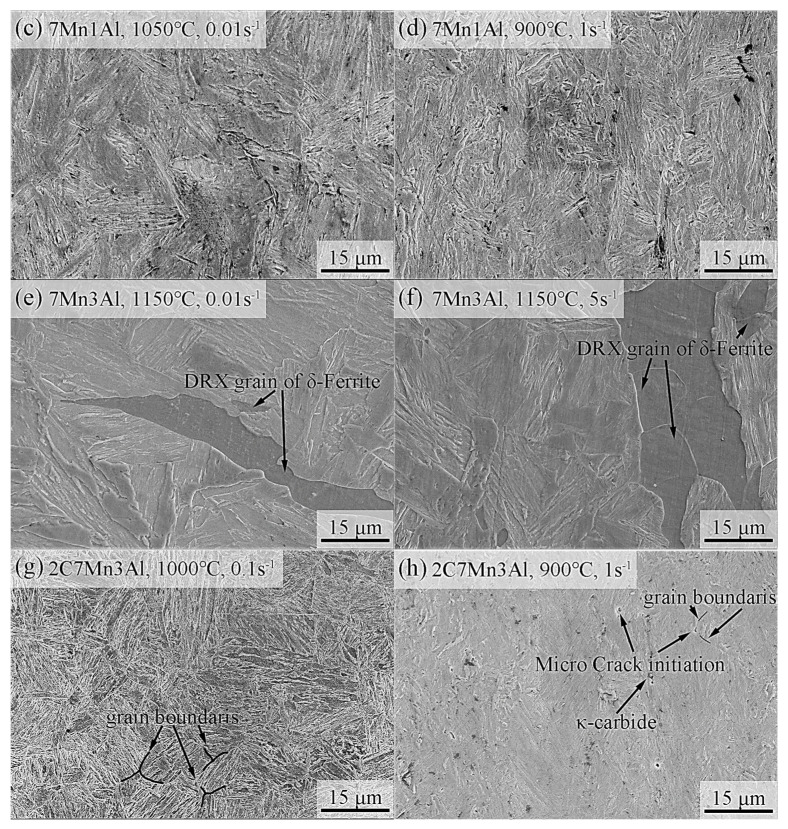
Microstructures of the steels under different hot working conditions: (**a**) 1150 °C, 0.01 s^−1^ and (**b**) 900 °C, 1 s^−1^ of 7Mn, (**c**) 1050 °C, 0.01 s^−1^ and (**d**) 900 °C, 1 s^−1^ of 7Mn1Al, (**e**) 1150 °C, 0.01 s^−1^ and (**f**) 1150 °C, 5 s^−1^ of 7Mn3Al, and (**g**) 1000 °C, 0.1 s^−1^ and (**h**) 900 °C, 1 s^−1^ of 2C7Mn3Al.

The microstructures of various steels subjected to different hot working conditions are depicted in Figure 8. Across all steels, the formation of martensitic microstructures during the cooling phase is evident. Figure 8a,b present the microstructures of 7Mn under varied hot working conditions, predominantly comprising martensite and retained austenite. However, the morphology and size of martensite differ based on distinct deformation processes. At 1150 °C and 0.01 s^−1^, martensite predominantly displays a lath-shaped structure. In contrast, at 900 °C and 1 s^−1^, two distinct forms—lath and block—are observed, with the former notably smaller in size compared with the latter.

In Figure 8c,d, the microstructure of 7Mn1Al predominantly reveals martensite. This observation indicates that the increase in Al content from 0 to 1.14 wt.% diminishes the stability of austenite, causing a complete transformation into martensite during the cooling phase. Concurrently, during hot deformation, the less stable austenite necessitates a lower critical strain to initiate dislocation slip, enhancing its propensity for plastic deformation. As a result, this enhancement potentially augments the hot-forming performance.

In Figure 8e,f, the microstructures of 7Mn3Al under different hot working conditions are depicted. Notably, distinct banded δ-ferrite is visibly apparent in the microstructure, consistent with the phase composition calculated at high temperatures using JMatPro. At this temperature, DRX is more prominently observed in δ-ferrite rather than in austenite. This disparity arises because the critical stress and strain required for DRX initiation in austenite exceed those in δ-ferrite [33]. In addition, compared with the sample with a strain rate of 5 s^−1^, inhomogeneous DRX grains were observed in the sample with a strain rate of 0.01 s^−1^, as shown in Figure 8e,f. This difference may be attributable to the lower strain rate, causing an earlier onset and prolonged duration of dynamic recrystallization, facilitating the comprehensive growth of DRX grains.

Upon comparison with 7Mn3Al, an evident reduction in the volume of δ-ferrite is observed in Figure 8g,h, when the carbon content increases from 0.03 wt.% to 0.2 wt.%. Additionally, the size of martensite laths notably diminishes in comparison to 7Mn3Al. Notably, carbides become apparent in the steel microstructure. Studies suggest that κ-carbide, composed of Fe, C, Al, and Mn, located at grain boundaries, may promote cracks, leading to quasi-cleavage or cleavage fracture modes [33]. Research indicates that carbides in medium-Mn steel might not favor microstructural stability [34]. The appearance of cracks dissipates a substantial amount of energy through plastic deformation, resulting in a lower *η* value in the corresponding region on the hot processing map under specific hot working conditions.

In summary, incorporating a specific quantity of Al can reduce the stability of austenite, thereby enhancing hot workability. However, an escalation in Al content can induce the appearance of δ-ferrite in the microstructure, consequently retarding the dynamic recrystallization of austenite and compromising hot workability. The addition of C serves to impede δ-ferrite formation, yet it triggers the formation of κ-carbide within the matrix. This occurrence renders the material susceptible to micro-crack formation at the precipitates, leading to intergranular fractures.

## 4. Conclusions

In this study, the hot deformation behavior of four types of medium-Mn steels with varying C and Al contents was examined through isothermal hot compression tests. The investigation focused on understanding the effects of elemental content on constitutive model parameters, hot workability, and microstructure. The findings are summarized as follows:

1. The relationship between the equilibrium phase content of the four steel variants and temperature was computed using JMatPro. At elevated temperatures, 7Mn and 7Mn1Al exhibit a single-phase structure of austenite, wherein the presence of Al elevates the complete austenitizing temperature. Conversely, 7Mn3Al and 2C7Mn3Al show a microstructure at high temperatures characterized by a mix of ferrite and austenite.
2. The flow stress of steel exhibits sensitivity to deformation temperature and strain rate, tending to rise with decreasing deformation temperature and increasing strain rate. The introduction of Al promotes ferrite formation in 7Mn3Al and 2C7Mn3Al at elevated temperatures. Uneven strain distribution between ferrite and austenite contributes to the elongation of the yield point in the initial stage of the flow stress curve. An Arrhenius constitutive model, incorporating Al content and strain compensation, was developed to predict the flow stress behavior in steel accurately.
3. The hot processing maps of the steels under 0.9 strain were established, and the 7Mn1Al exhibits the best hot workability. The optimum hot working interval is deformation temperature 975–1125 °C, strain rate 0.2–5 s^−1^.
4. The rise in Al content from 0 to 1.14 wt.% diminished the stability of austenite, enhancing the hot processing capabilities of steel. However, when the content reached 3.3 wt.%, the emergence of δ-ferrite in the microstructure hindered austenite DRX, negatively affecting hot workability. The addition of C reduces the volume of δ-ferrite but results in κ-carbide precipitation, leading to micro-crack formation, which adversely affects processing.

## Figures and Tables

**Table 1:** Chemical composition of 7Mn steels (wt.%).

Steels	C	Mn	Al	Cr	Ni	Mo
7Mn	0.01	6.19	0.00	0.16	0.59	0.03
7Mn1Al	0.02	6.53	1.14	0.13	0.11	0.06
7Mn3Al	0.03	6.78	3.30	0.12	0.04	0.13
2C7Mn3Al	0.20	6.58	3.46	0.12	0.03	0.14

**Table 2:** Constitutive model-related constants of Al-containing medium-Mn steels.

Coefficients	G			
	α	n	Q (KJ/mol)	lnA
*k*_0_	1.69 × 10^−2^	7.64	4.02 × 10^5^	3.50 × 10^1^
*a*_1_	−1.73 × 10^−3^	4.80 × 10^−1^	−1.89 × 10^4^	−1.93
*a*_2_	1.09 × 10^−4^	−1.16 × 10^−1^	1.75 × 10^3^	0.22
*a*_3_	2.04 × 10^−4^	−1.37 × 10^−2^	6.79 × 10^3^	0.64
*a*_4_	−3.71 × 10^−5^	5.09 × 10^−3^	−1.20 × 10^3^	−0.11
*b*_1_	−2.32 × 10^−2^	−6.23	6.38 × 10^5^	4.53 × 10^1^
*b*_2_	−1.77 × 10^−1^	−5.00 × 10^1^	−1.08 × 10^7^	−8.36 × 10^2^
*b*_3_	1.62	3.11 × 10^2^	6.34 × 10^7^	5.03 × 10^3^
*b*_4_	−5.00	−8.82 × 10^2^	−1.91 × 10^8^	−1.54 × 10^4^
*b*_5_	7.59	1.36 × 10^3^	3.02 × 10^8^	2.48 × 10^4^
*b*_6_	−5.68	−1.06 × 10^3^	−2.40 × 10^8^	−1.98 × 10^4^
*b*_7_	1.68	3.29 × 10^2^	7.47 × 10^7^	6.21 × 10^3^

## Data Availability

Data are contained within the article.
